# Rheology-Informed Neural Networks (RhINNs) for forward and inverse metamodelling of complex fluids

**DOI:** 10.1038/s41598-021-91518-3

**Published:** 2021-06-08

**Authors:** Mohammadamin Mahmoudabadbozchelou, Safa Jamali

**Affiliations:** Department of Mechanical and Industrial Engineering, Northeastern University, Boston, MA 02115 USA

**Keywords:** Engineering, Chemical engineering, Mechanical engineering, Materials science, Soft materials, Theory and computation

## Abstract

Reliable and accurate prediction of complex fluids’ response under flow is of great interest across many disciplines, from biological systems to virtually all soft materials. The challenge is to solve non-trivial time and rate dependent constitutive equations to describe these structured fluids under various flow protocols. We present Rheology-Informed Neural Networks (RhINNs) for solving systems of Ordinary Differential Equations (ODEs) adopted for complex fluids. The proposed RhINNs are employed to solve the constitutive models with multiple ODEs by benefiting from Automatic Differentiation in neural networks. In a direct solution, the RhINNs platform accurately predicts the fully resolved solution of constitutive equations for a Thixotropic-Elasto-Visco-Plastic (TEVP) complex fluid for a series of flow protocols. From a practical perspective, an exhaustive list of experiments are required to identify model parameters for a multi-variant constitutive TEVP model. RhINNs are found to learn these non-trivial model parameters for a complex material using a single flow protocol, enabling accurate modeling with limited number of experiments and at an unprecedented rate. We also show the RhINNs are not limited to a specific model and can be extended to include various models and recover complex manifestations of kinematic heterogeneities and transient shear banding of thixotropic fluids.

## Introduction

Complex fluids are a broad class of materials, in which the macroscopic response of the fluid to an applied deformation or load is determined by the state of microstructure. In contrast to conventional fluid mechanics problems, where the viscosity of the fluid remains constant, the material functions of the complex fluids depend on the rate and time of applied deformation^1–10^. To predict these complex fluids’ behavior under flowing conditions, it is indispensable to present closed-form constitutive equations that correlate the microstructural and kinematic variables of the material to the state of stress. Efforts in developing such constitutive equations are thus as old as the science of rheology itself^11–13^. The constitutive models of choice become more intricate, as the fluid’s response to a deformation becomes rate or time dependent, leading to an inevitable increase in the number of model parameters. Hence, more experimental protocols are needed to determine these parameters and to describe the system under question. Nonetheless, even constitutive equations with several model parameters commonly fail to capture the rheology of a complex system subject to a series of different flow protocols.

Complex fluids often exhibit a time-dependent stress response under flow owing to their inherent viscoelastic and/or thixotropic timescales^14–16^. Thixotropy observed in many complex fluids generally manifests in the sensitivity of the viscosity to the history of the applied strain rate^17–19^. Thixotropic effects originate from evolution of the material’s microstructure as a result of the interplay between shearing forces exerted by the flow and the natural structure formation^20–22^. Thus, in thixotropic constitutive equations, one will critically need to solve for the time evolution of a structure parameter under flow. On the other hand, the local shear stress/rate that the material experiences determines the rate of structure break-up under flow. Hence, detailed multi-component constitutive models that fully capture different rate and time dependent phenomena commonly involve systems of coupled differential equations. Many constitutive models have been proposed to recover thixotropic response of a complex fluid^23–27^. For an ideal thixtropic fluid, also referred to as Thixo-Visco-Plastic (TVP) fluid, the shear stress depends on the structure parameter, $\lambda$, which itself evolves with time as shown in Eq. (1)^23^. In this equation, $\sigma _y$ is the yield stress, $\eta _s$ and $\eta _p$ are background and plastic viscosities, ${\dot{\gamma }}$ is the applied deformation rate, and $k_+$ and $k_-$ are the build-up and breakage coefficients of the structure parameter.

1 $$\begin{aligned} \begin{aligned} {\left\{ \begin{array}{ll} \sigma (t)&{}= \sigma _y \lambda (t) + (\eta _s + \eta _p \lambda (t)){\dot{\gamma }}(t) \\ {\dot{\lambda }}(t) &{}= k_+ (1 - \lambda (t)) - k_- \lambda (t) {\dot{\gamma }} (t) \end{array}\right. } \end{aligned} \end{aligned}$$

Colloidal gels commonly show thixotropic, static and dynamic yielding, rate-dependent shear thinning, and elsatic response under different flow protocols and are referred to as Thixotropic Elasto-Visco-Plastic (TEVP) fluids^3,28–31^. Thus, in addition to TVP model parameters, TEVP constitutive equations include the elastic modulus (*G*) of the fluid as well. A TEVP model is shown in Eq. (2), including six different model parameters.

2 $$\begin{aligned} \begin{aligned} {\left\{ \begin{array}{ll} {\dot{\sigma }}(t) &{}= \frac{G}{\eta _s + \eta _p}[-\sigma (t) +\sigma _y \lambda (t)+ (\eta _s + \eta _p \lambda (t)){\dot{\gamma }}(t)] \\ {\dot{\lambda }}(t) &{}= k_+ (1 - \lambda (t)) - k_- \lambda (t) {\dot{\gamma }}(t) \end{array}\right. } \end{aligned} \end{aligned}$$

While the model presented in Eq. (2) recovers a number of rheological features of TEVP fluids, in order to fully capture the response of the fluid to a Large Amplitude Oscillatory Shear (LAOS) flow protocol, a more sophisticated plastic component has to be considered. Iso-Kinematic Hardening (IKH) model^4,32^ decouples the applied shear rate into plastic and viscoelastic contributions and introduces the back strain in order to account for the evolving microstructure from one cycle to next in oscillatory flows. This leads to a complex constitutive equation that predictably captures wide range of material behavior with different flow protocols. The general description of IKH model is shown as Eq. (3). The function *f*(.) is determined based on the viscoelastic model of choice that leads to acquisition of various models consisting of 9-15 parameters that are inevitably challenging to be determined. In this set of coupled ODEs, *A* is the back strain, *m* and *q* are the material constants, and ${\dot{\gamma }}_p$ is the plastic component of the applied shear rate.

3 $$\begin{aligned} \begin{aligned} {\left\{ \begin{array}{ll} {\dot{\sigma }}(t) &{}= f(\sigma ,C,A(t),\lambda (t),{\dot{\gamma }}(t)) \\ {\dot{A}}(t) &{}= \dot{\gamma _p} - (q|A(t)|)^m sign(A(t))|\dot{\gamma _p}(t)| \\ {\dot{\lambda }}(t) &{}= k_+ (1 - \lambda (t)) - k_- \lambda (t) |\dot{\gamma _p}(t)| \end{array}\right. } \end{aligned} \end{aligned}$$

As is clearly evident in Eq. (3), the number of parameters required to fully capture the rheological response of a complex fluid to an applied deformation increases very rapidly and eventually becomes computationally prohibitive. Moreover, these parameters are not necessarily based on a physical merit, and are often challenging to fit. Thus, an exhaustive list of experimental protocols are usually taken to fully parametrize a given model for a specific system. Even then, the emergence of multiple length and time scales due to structure break-up/formation, non-ideal behavior of the material under investigation, experimental artifacts, and many more delicate details can lead to erroneous predictions. This is even more evident when real-life and industrial complex fluids of interest that contain multiple components are considered. Thus, numerical platforms that reduce the computational complexity of implementing a fully resolved constitutive model, or decrease the number of experiments required to identify a system’s model parameters are of great interest.

Over the past few years, Machine Learning (ML) algorithms have found their way in all avenues of science and engineering. With an ever-increasing computational power and the ability to process large data sets, data-driven models have become indisputable and powerful tools. With a limited number of studies utilizing ML algorithms^33–36^, the field of soft matter and more specifically rheology is lagging behind in leveraging such advanced methodologies. This is partially due to the ambiguous consequences of the produced meta-models and their adherence to the fundamental underlying physics. However, these issues would be effectively attenuated by executing the appropriate type of ML approach.

Traditional ML algorithms, regardless of their type, depend on abundance of data to be accurately predictive. This means it is absolutely essential to train the considered ML algorithms on extremely large enough data set. Moreover, most of ML algorithms are suitable for interpolation [when they are trained on a sufficiently large data sets], and are often incapable of out-of-range predictions (extrapolation). Recent physics-based ML algorithms not only include the physical governing equations of choice, but also diminish the need for big data sets. The groundbreaking work of Raissi et al.^37^ on “Physics-Informed Neural Network” (PINN) paved the way for physics-based ML algorithms to address these issues. The central concept is to directly add physical governing equations to the neural network (NN) framework to achieve a meaningful meta-model. By incorporating the governing physical laws, and constraining the NN framework to adhere to these physical laws, the need for large training data sets can also be eliminated. It is worth mentioning that the scope of this work is limited to problems in which the constitutive model describing the material of choice is known. In the case with unknown governing laws, the pathway to embedding the physical laws into the training process has to change accordingly. One such method would be to introduce the physical intuition to the NN implicitly and by means of physics-based synthetic data, generated from constitutive laws^33^.

In this study, we present Rheology-Informed Neural Networks (RhINNs) for direct and inverse solution of complex rheological constitutive models. In a direct solution, RhINNs are employed as an alternative platform for solving systems of Ordinary Differential Equations (ODEs) on predicting the rheology of complex fluids. In the inverse solution however, RhINNs are used to learn the hidden rheology of complex fluids with only a handful of data sets. To this end, we first describe the meta modeling approach using NNs in the form of RhINNs. Thereupon, results are presented for both direct and inverse problems, followed by concluding remarks and outlooks.

## Problem setup and methodology

**Figure 1:**
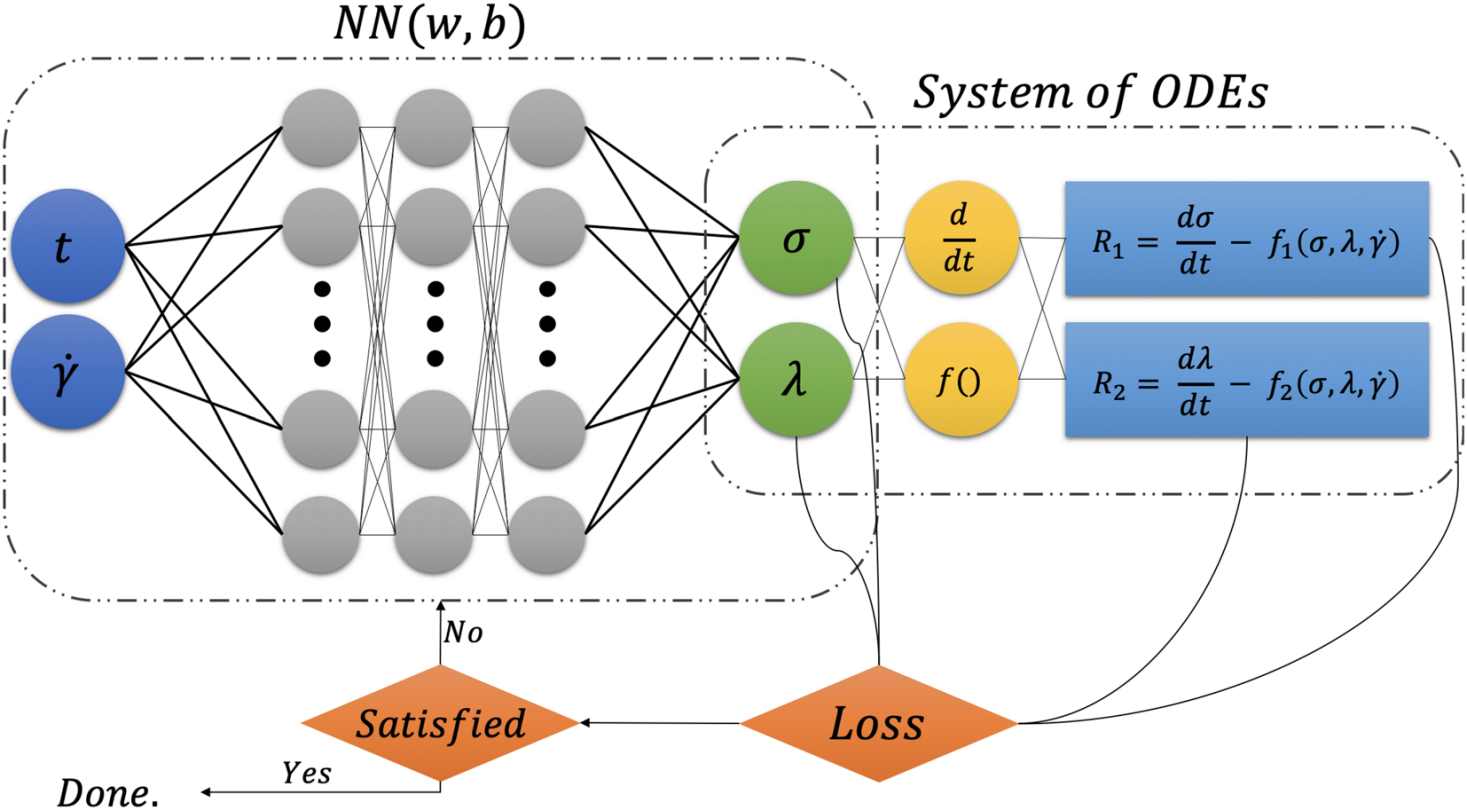
The Schematic architecture of a rheology-informed neural network (RhINNs). For visual purposes, a plain vanilla NN with three hidden layers and four neurons per layer is shown. As a motivating example, if a TEVP material described by Eq. (2) is included in the network, the definitions of $f_i()$ would turn to $f_1(\sigma ,\lambda ,{\dot{\gamma }})=\frac{G}{\eta _s + \eta _p}[-\sigma (t) +\sigma _y \lambda (t)+ (\eta _s + \eta _p \lambda (t)){\dot{\gamma }}(t)]$ and $f_2(\sigma ,\lambda ,{\dot{\gamma }})=k_+ (1 - \lambda (t)) - k_- \lambda (t) {\dot{\gamma }}(t)$.

Neural Networks are a sub-class of supervised ML algorithms^38^, consisting of many interconnected processing elements called neurons. Neurons process and predict data by creating a computational structured framework where the complex relations between the inputs and outputs is revealed as a function. Each NN consists of three main layers: input layer, output layer, and several hidden layers. Each of these hidden layer contains several neurons, and each neuron has an specific weight and bias. These networks learn to minimize their deviations from the actual data by adjusting the weights and biases between different neurons and layers within the structure of the network. In other words, the weights and biases of the neurons are changed continuously to generate an emend response when new inputs are provided. NNs generate meta-models based on these correlations in statistical variations of complex systems. In a purely statistical method, the training process for the NN is agnostic to the physical governing equations. Here however, we directly solve for nonlinear problem without any prior assumptions, linearization, or local time-stepping. We benefit from recent developments in automatic differentiation^39^ to differentiate the NN with respect to its input coordinates and model parameters. In other words, we include the physical laws explicitly into the NN architecture. Figure 1 shows a schematic description of RhINNs. For visual purposes, Fig. 1 contains a NN with only three hidden layers and four neurons per layer, with 2 input parameters as time (*t*) and shear rate $({\dot{\gamma }})$, and 2 output parameters as shear stress $(\sigma )$ and structure parameter $(\lambda )$. We should mention that in the definition of $f_i()$, shear rate as ${\dot{\gamma }}$ plays an important role, since it reflects on the kinematics of the imposed flow protocol. Hence, including such function is a necessity either implicitly (as a function in the physical governing law) or explicitly (as one of the inputs of RhINNs). We chose to go with the latter, since we are offering a more generic framework without affecting the predicted results. We performed a comprehensive analysis to determine the effects of number of hidden layers and number of neurons in each hidden layer on the performance of our proposed RhINNs, which are presented in Appendix B.

In a data driven solution framework, the solution of the constitutive equation of choice is being inferred without any data, and the only thing that is needed is the constitutive equation itself and the initial conditions to the problem of interest. In this framework, one can think of the NN as an alternative ODE or PDE solver, where inputs are correlated directly to the predictions. These inputs and their corresponding predictions are used to calculate the residual of the constitutive model at hand, and the goal of the NN is to minimize this residual. Only then one can assure that the training process is informed by a physical intuition. On the other hand and in a data driven discovery framework, the input of the RhINNs is a $n \times 3$ matrix, in which the first and second columns are time and shear rate (${\dot{\gamma }}$), respectively, and the final column is the shear stress at that particular shear rate and time measured experimentally or calculated numerically. Since experimental observation of structure parameter is not feasible, we cannot include this information into the training process. Here by knowing the experimental measurements of only shear stress, the goal of NN is to minimize the residual for the constitutive model of choice and return the predicted model parameters.

In general, a system of ODEs with two independent variables can be written as Eq. (4).

4 $$\begin{aligned} \begin{aligned} {\left\{ \begin{array}{ll} \dot{y_1} &{}= {\mathcal {F}}_1(y_1,y_2,t) \\ \dot{y_2} &{}= {\mathcal {F}}_2(y_1,y_2,t) \end{array}\right. } \end{aligned} \end{aligned}$$

In this system of ODEs, $y_1(t)$ and $y_2(t)$ are the hidden solution and ${\mathcal {F}}_i$ are nonlinear operators in the time domain of [0, *T*]. As a motivating example, a TEVP material [described by Eq. (2)], represents a system of ODEs with two equations. Hence, the definitions of $f_i()$ shown on the right box of Fig. 1 would turn into $f_1(\sigma ,\lambda ,{\dot{\gamma }})=\frac{G}{\eta _s + \eta _p}[-\sigma (t) +\sigma _y \lambda (t)+ (\eta _s + \eta _p \lambda (t)){\dot{\gamma }}(t)]$ and $f_2(\sigma ,\lambda ,{\dot{\gamma }})=k_+ (1 - \lambda (t)) - k_- \lambda (t) {\dot{\gamma }}(t)$. By adjusting the correspondence from one neuron to another, and from one layer to another in a NN, a meta-model is produced to correlate the output results based on a series of new input variables. The variables of a RhINNs are learned by minimizing the loss function, that captures the residual of each equation in addition to the the discrepancy between the predicted and the actual Initial Condition (IC) during the training process. Eqs. (5) and (6) present the RhINNs loss functions for the direct and the inverse problems, respectively.

5 $$\begin{aligned} MSE_{Dir}= & {} MSE_{R} + MSE_{IC} \end{aligned}$$

6 $$\begin{aligned} MSE_{Inv}= & {} MSE_{R} + MSE_{d} \end{aligned}$$

In our system, and in Eqs. (5) and (6), $MSE_{R}$ (Eq. 7) is the residual calculated from the system of ODEs, $MSE_{d}$ (Eq. 8) is the deviation of RhINNs predictions from actual values , and $MSE_{IC}$ (Eq. 9) is discrepancy between the actual and the predicted values of the initial conditions. In practice, initial conditions are imposed by calculating the predicted output at t=0 and adding the discrepancy between the predicted value and actual initial condition as defined in Eq. (8) to total loss function. It should be noted that in an inverse approach, existence of IC is not a necessity.

7 $$\begin{aligned} MSE_{R}= & {} \sum _{j=1}^{N_{eqs}} \frac{1}{N_{R_j}} \sum _{i=1}^{N_{R_j}} |Residual_{(equation_j)}(t_i)|^2 \end{aligned}$$

8 $$\begin{aligned} MSE_{IC}= & {} |Predicted_{IC}-Actual_{IC}|^2 \end{aligned}$$

9 $$\begin{aligned} MSE_{d}= & {} \sum _{i=1}^{N_d} |Predicted(t_i)-Actual(t_i)|^2 \end{aligned}$$

In an inverse problem, the model parameters are chosen to be variables that can be changed throughout the optimization process. After initialization, a total loss is calculated based on Eq. (6). Afterward, these variables are consistently changed during the optimization process until the loss function is minimized (and becomes zero in an ideal case). After reaching a certain criteria, the training process stops and the model parameters are presented. It should be mentioned that there are no strict boundaries set for any of the parameters used in this work.

## Results and discussion

As describe previously, the ultimate goal is to develop a reliable and accurate platform for fast data-driven solution of complex time and rate dependent constitutive equations. Thus here, the scope of our study is limited to demonstrating RhINNs as a robust alternative meta-constitutive model. In the following, several flow protocols of rheometric significance are solved in both direct and inverse problems, referred to as data driven solution and data driven discovery respectively. In data driven solution the NN is employed to find an answer in a certain domain for an existing set of equations and initial conditions. On the other hand, with the inverse problems, i.e. data driven discovery, the characteristics of a system of ODEs and hence material’s properties are predicted using the data at hand and the system of ODEs.

### Data driven solution

**Figure 2:**
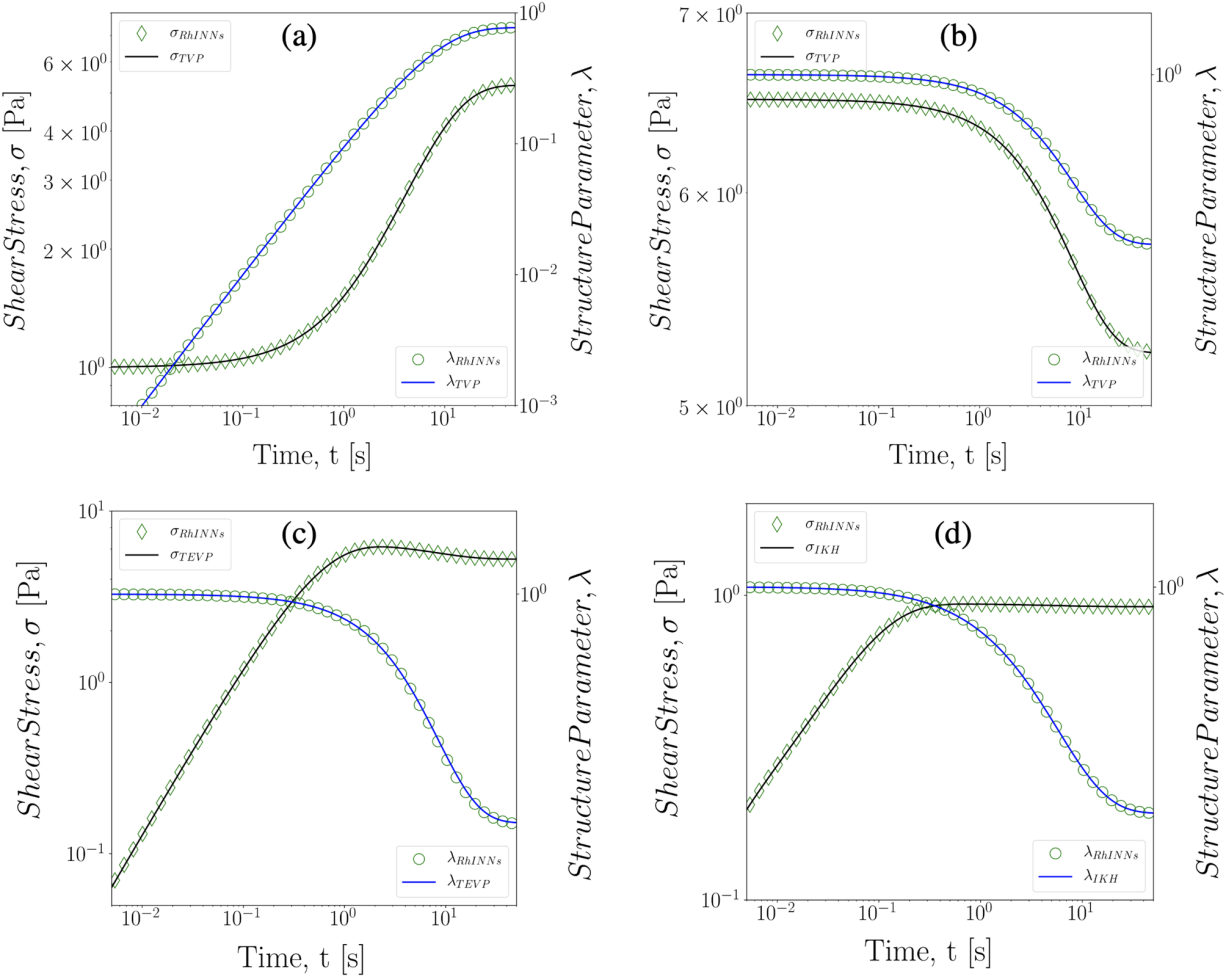
Direct solution of different constitutive models in a shear start-up flow protocol and applied shear rate of $0.1\,[1/\text {s}]$: *(a)* Fully fluidized TVP fluid, *(b)* fully structures TVP fluid , *(c)* fully fluidized TEVP, and *(d)* fully fluidized TEVP fluid with IKH model, with parameters based on Table 1.

RhINNs are devised and employed as alternative tools to solve systems of ODEs used in complex fluid modelling. In the training process, only the system of ODEs and the initial conditions are used without any additional data, hence the output of the NN will be the solution to the constitutive model. First, we consider different models outlined in Eqs. (1), (2), and (3) to show the capability of RhINNs in solving various constitutive equations. Then, we explore the role of rheometric protocol by solving for the stress response under a range of different deformation protocols. Note that there exist a number of viable options as thixotropic constitutive models to be adapted here; however, we are considering the three different constitutive models in Eqs. (1), (2), and (3), as they provide an increasing level of complexity with considerable number of model parameters involved in the IKH model: Eq. (1) is the simplest model for a thixotropic material with five (5) model parameters and a single algebraic equation coupled with an ODE, Eq. (2) includes elasticity with an additional parameter, and Eq. (3) contains a total of nine (9) model parameters and three coupled equations. Figure 2 shows the comparison between the ground truth solution of different thixotropic constitutive models and RhINNs predictions with parameters based on Table 1 and in a start-up of shear flow protocol with ${\dot{\gamma }}=0.1\,\text {s}^{-1}$. While Table 1 outline the choice of model parameters used in Fig. 2 for each model, we performed similar benchmarking with a wide range of parameters and initial conditions, and found that the RhINNs predictions are not limited by the choice of parameters or the initial conditions. Results in Fig. 2 clearly indicate that RhINNs’ predictions closely track the ground solution of the shear stress response, regardless of the choice of model. The value of the microstructure parameter, $\lambda$, ranges from zero for a fully destructured/fluidized system, to unity for a fully structured material, ex. unyielded gel. Comparing the Fig. 2a,b, where fully fluidized and fully structured systems are compared, it is evident that the RhINNs predictions remain valid by changing the initial conditions as well. The Fig. 2c,d respectively show the RhINNs-predicted flow curve as well as the ground truth solution of TEVP, and IKH constitutive models, with increasing levels of complexity.

**Table 1:** Values of the model parameters used for the flow curves presented in Fig. 2.

	$G\,[\text {Pa}]$	$s_y\,[\text {Pa}]$	$\eta _s\,[\text {Pa s}]$	$\eta _p\,[\text {Pa s}]$	$k_p\,[1/\text {s}]$	$k_n$	$C\,[\text {Pa}]$	*q*	*m*
TVP	–	5	10	5	0.1	0.3	–	–	–
TEVP	30	5	10	5	0.1	0.3	–	–	–
IKH	100	0.1	500	1.4	0.1	0.3	70	100	0.5

**Figure 3:**
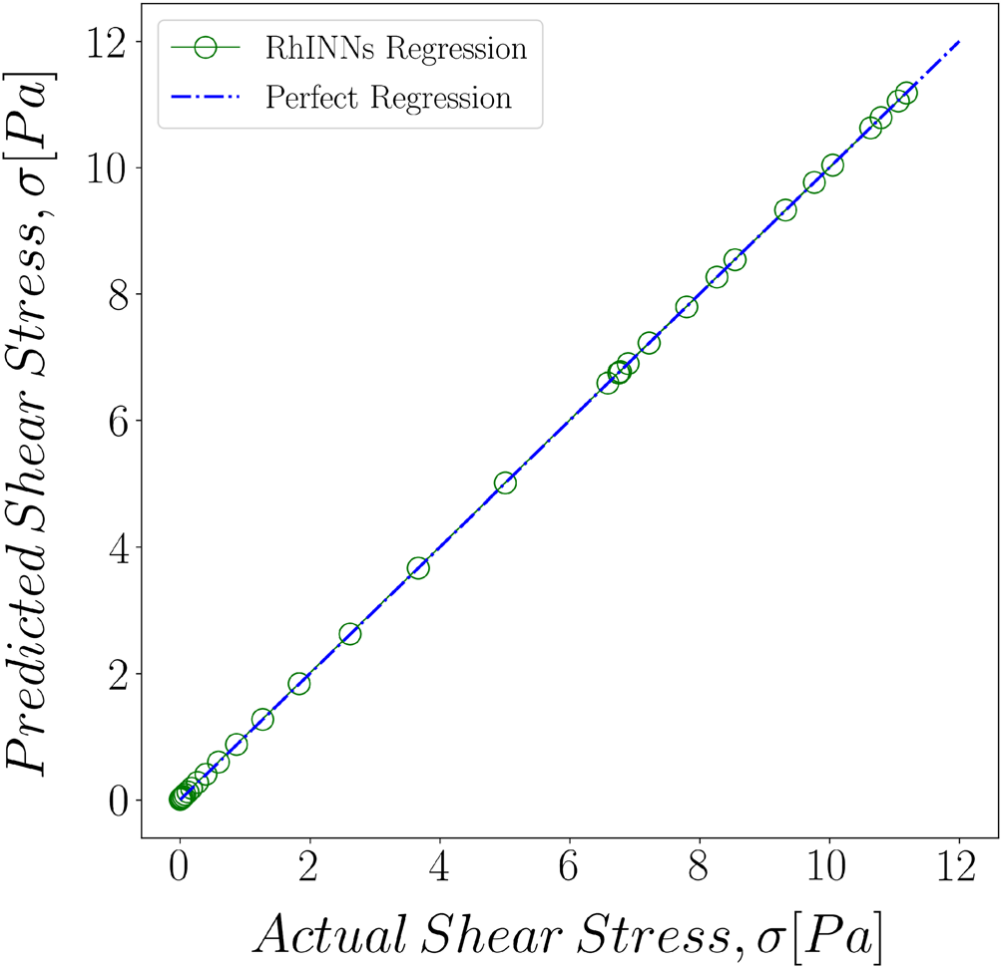
The regression between the ground solution and the predictions made by RhINNs for a direct problem based on a TEVP mode in start-up of flow with the shear rate of ${\dot{\gamma }}=0.1\,[1/\text {s}]$.

The regression plot of the trained model for a direct problem with a TEVP model at the shear rate of ${\dot{\gamma }}=0.1\,[1/\text {s}]$ is shown in Fig. 3. As the figure shows, there is an excellent correlation between the predicted solution and the ground solution in this case, suggesting that the training is performed properly.

**Figure 4:**
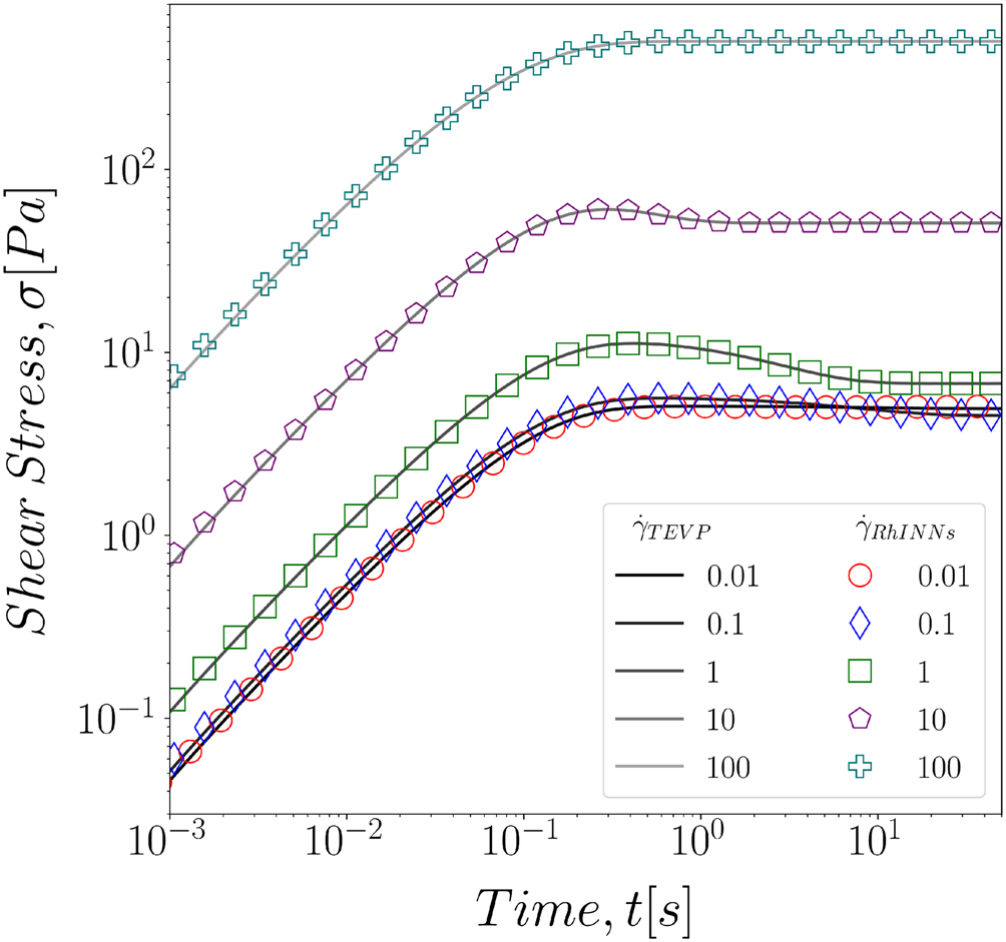
Direct RhINNs solution of a TEVP fluid under constant shear rate flow start-up protocol, with deformation rates ranging from ${\dot{\gamma }}=0.01{-}100\,\text {s}^{-1}$ . The TEVP model parameters are: $G = 70\,[\text {Pa}]$, $\sigma _y = 5\,[\text {Pa}]$, $\eta _s = 5\,[\text {Pa s}]$, $\eta _p = 2\,[\text {Pa\,s}]$, $k_+ = 0.1\,[1/\text {s}]$, $k_- = 0.3$.

In the next step, we sought to investigate the role of the shear rate magnitude on the RhINNs predictions for the stress response of TEVP fluids. This is particularly important with respect to application of any data driven methodology to rheometric flows and predictions, where differences in the magnitude of applied rates and resulting stresses are commonly presented in logarithmic scales. Since the yield stress and the steady state shear stress, depending on the applied deformation rate, can greatly differ in their magnitude, it is critical to ensure that the neural network provides a reliable prediction for the low shear stress regime and the high shear stress regime alike. In other words, one has to ensure that the small values of stress and the residuals for the correlations in this shear regime are not screened by the large stresses at the highest deformation rates. To do this, we consider the Eq. (2) to be the constitutive model of choice with simple start up of shear protocol. Five different shear rates from ${\dot{\gamma }}=0.01\,\text {s}^{-1}$ to ${\dot{\gamma }}=100\,\text {s}^{-1}$ are presented to cover four decades of change in shear rate. As presented in Fig. 4 the predictions made by the RhINNs and the ground solution of the TEVP fall exactly on top of one another for all shear rates studied here.

**Figure 5:**
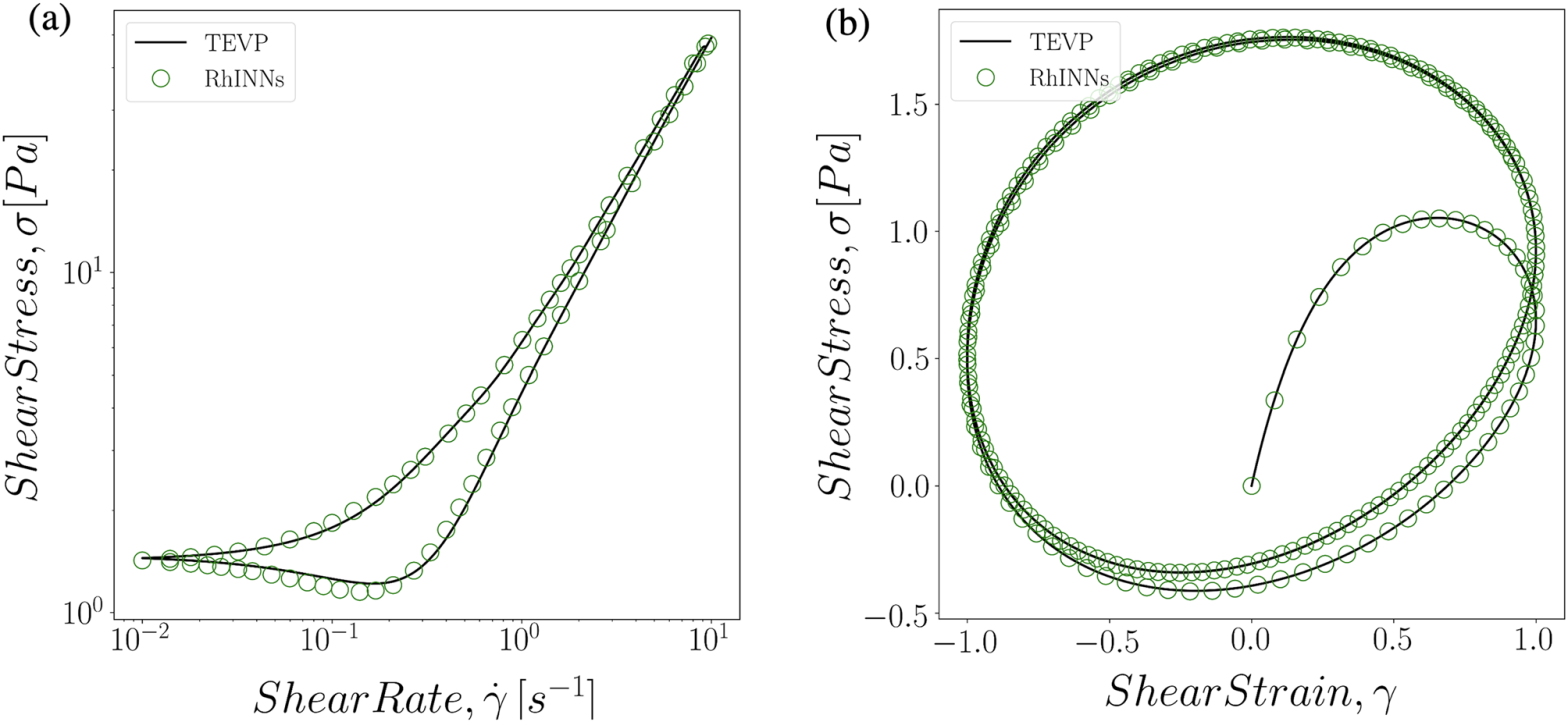
Direct RhINNs solution of a TEVP constitutive equation with two different flow protocols: *(a)* ramp function with the deformation rate starting from 10 [1/s] and linearly decaying to 0.01 [1/s] over 50 s, followed by increases to the initial value over the same period of time. And *(b)* Large Amplitude Oscillatory Shear (LAOS) flow with unit amplitude and frequency of $\omega = 0.2$. For both protocols the model parameters are: $G = 5.5\,[\text {Pa}]$, $\sigma _y = 1\,[\text {Pa}]$, $\eta _s = 5\,[\text {Pa s}]$, $\eta _p = 0.5 [Pa.s]$, $k_+ = 0.1\,[1/\text {s}]$, $k_- = 0.3$.

In practice, a number of different flow protocols are commonly applied to a complex fluid to probe the relevant material function, properties and characteristic timescales. Start-up of shear, flow hysteresis or ramp cycles, small amplitude oscillatory shear (SAOS), LAOS, and step shear rate are among the most common rheometric flow protocols that can be used in order to fully investigate a thixotropic fluid. The data-driven methodologies commonly fail to capture the details of changes in a flow protocol since the equations are not fully solved, but are merely correlated in time. The traditional data-driven methodologies, such as deep neural networks without introduction of physical laws, commonly fail to capture the details of changes in a flow protocol since the equations are not fully solved, but are merely correlated in time. For instance, even if enough data is used for accurate training of a deep neural network for constant shear rate flow protocol, the network learns to predict the steady state response of the material to an applied deformation rate at long time (longer than the material timescale). Thus, when a flow protocol involves change of direction or magnitude at a later time, classical neural networks lose their ability to track the experiment entirely. Nonetheless, RhINNs does not suffer from the same deficiency and is able to recover these rate changes in different protocols. Figure 5 shows the comparison between the RhINNs prediction and the ground solution of the shear stress response of a TEVP fluid (Eq. 2) under flow hysteresis and LAOS experiments. The rheological hysteresis area is a hallmark of thixotropic fluids, where a ramp down followed by a ramp up shear protocol, returning to the initial shear rate (which is large enough to fluidize the entire system and erase any thermokinematic memory) results in close-loop flow curves. The physical significance of such protocols is the fact that the magnitude of this area strictly depends on the characteristic timescale at which the material begins to erase its memory to the previous deformation. Hence, such methods are used commonly to characterize the thixotropic timescale in TEVP fluids^20,21^. On the other hand, the so-called Lissajous curves that describe the shear stress response of a fluid to a large oscillatory shear deformation have been studied extensively in order to characterize time and rate dependent complex fluids such as TEVPs^4,26,32,40–43^. In both protocols, RhINNs closely mimics the ground solutions of the TEVP constitutive model over the entire range of shear rates and amplitudes (for brevity, only one frequency and amplitude is presented).

**Figure 6:**
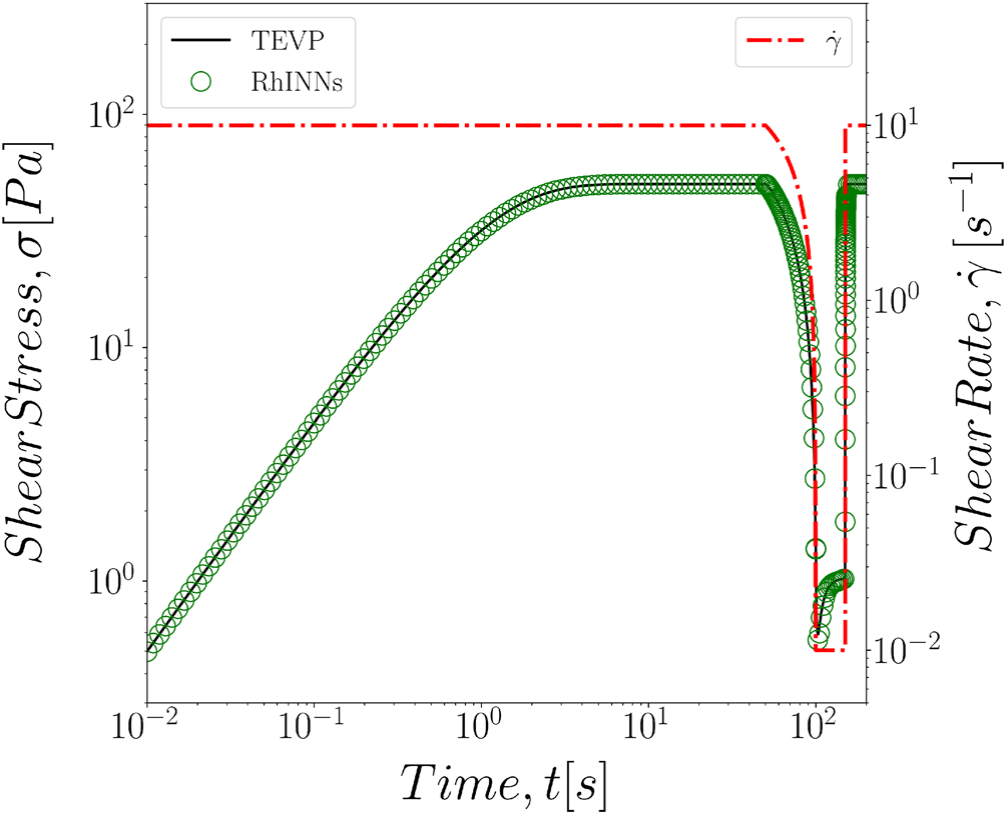
Direct RhINNs solution of a TEVP fluid’s stress response to a multi shear rate protocol. The model parameters are similar to ones in Fig. 5.

In order to fully probe the ability of our RhINNs methodology to predict the stress response of a TEVP fluid to temporal changes in the imposed shear rate, a more complex step rate-change protocol was applied. Figure 6 represents the comparison between the RhINNs predictions and the ground solutions of the TEVP constitutive model for the shear stress response of a complex shear rate protocol applied to the complex fluid: initial shear rate of ${\dot{\gamma }}=100\,\text {s}^{-1}$ is applied for 50 s, followed by a linear ramp down to ${\dot{\gamma }}=0.1\,\text {s}^{-1}$ over the next 50 s. Upon reaching ${\dot{\gamma }}=0.1\,\text {s}^{-1}$ the deformation rate is kept constant for the third 50 s of the protocol, followed by a final step-up to the initial shear rate of ${\dot{\gamma }}=100\,\text {s}^{-1}$. The results in Fig. 6 clearly indicate that even with a complex shear rate protocol, RhINNs gives a robust predictions with virtually no deviation from the ground solution of the constitutive equation.

### Data driven discovery

As described previously, a major leap forward in constitutive modelling of complex fluids and in material design and discovery can be made by enabling data driven methods that recover material functions from a limited number of experiments. Practically, a series of different experiments are performed in order to fit a particular model that describes observed rheological behavior, in order to determine the model parameters and hence material properties of a thixotropic fluid. Of particular interest is to determine the timescales and kinetics of structure break-up and formation under different flowing conditions. Thus in this section, we seek to find the model parameters and material’s time constants from a series of simple flow curves. To do this, we employ our RhINNs methodology to solve for the inverse problem, and predict the material parameters from the shear stress response, i.e. find the hidden rheology with a limited set of data. This is done pedagogically, and by beginning with an assumption of partial information regarding material properties. The ultimate goal is to identify the number of experiments required to provide an accurate prediction for the material properties using the inverse RhINNs platform. These properties include the elastic modulus and the yield stress of the fluid, as well as the time constants required to recover the temporal evolution of the structure parameter. The number of data points commonly collected over a particular rheometric flow protocol greatly depends on the material under investigation, the inherent timescales associated with the material and with the flow protocol, etc.. Nonetheless, typically rheological data are collected and represented in logarithmically spaced intervals to reveal the material functions/timescales with respect to the applied flow protocol. Here, we used $\sim 200$ data points to remain in a relevant data size with respect to the common experiments. In the first step, we seek to identify the time constants for the time evolution of the structure parameter from the shear stress response, assuming that the yield stress and the elastic modulus of the fluid are known. Namely, flow curves such as ones presented in Fig. 2 are provided, and the RhINNs is used to recover the model parameters. Table 2 represents the actual and predicted values of $k_+$ and $k_-$, using a single shear flow curve, whether that is start up of flow, step rate, ramp cycle or SAOS/LOAS. For all various flow protocols, RhINNs recovers the time constants for the structure evolution, having the rest of parameters, with less than one percent error. The exception is the oscillatory shear protocol, for which the error rises to smaller than 5 percent. Nonetheless, this is extremely accurate considering that for each of these protocols, only one set of shear stress response is provided for RhINNs to learn the hidden rheology.

**Table 2:** RhINNs-predicted values for $k_+$ and $k_-$ in different shear rate protocols. For all cases $G = 30\,[\text {Pa}]$, $\sigma _y = 0.5\,[\text {Pa}]$, $\eta _s = 5\,[\text {Pa s}]$, and $\eta _p = 1\,[\text {Pa s}]$ are known from the material properties.

	Actual value	Predicted value			
		Simple shear	Step rate	Ramp cycle	SAOS
$k_-$	0.300	0.301	0.300	0.303	0.286
% Error	–	0.46	0.09	0.99	4.52
$k_+$	0.100	0.100	0.100	0.101	0.097
% Error	–	0.38	0.05	0.81	3.32

**Figure 7:**
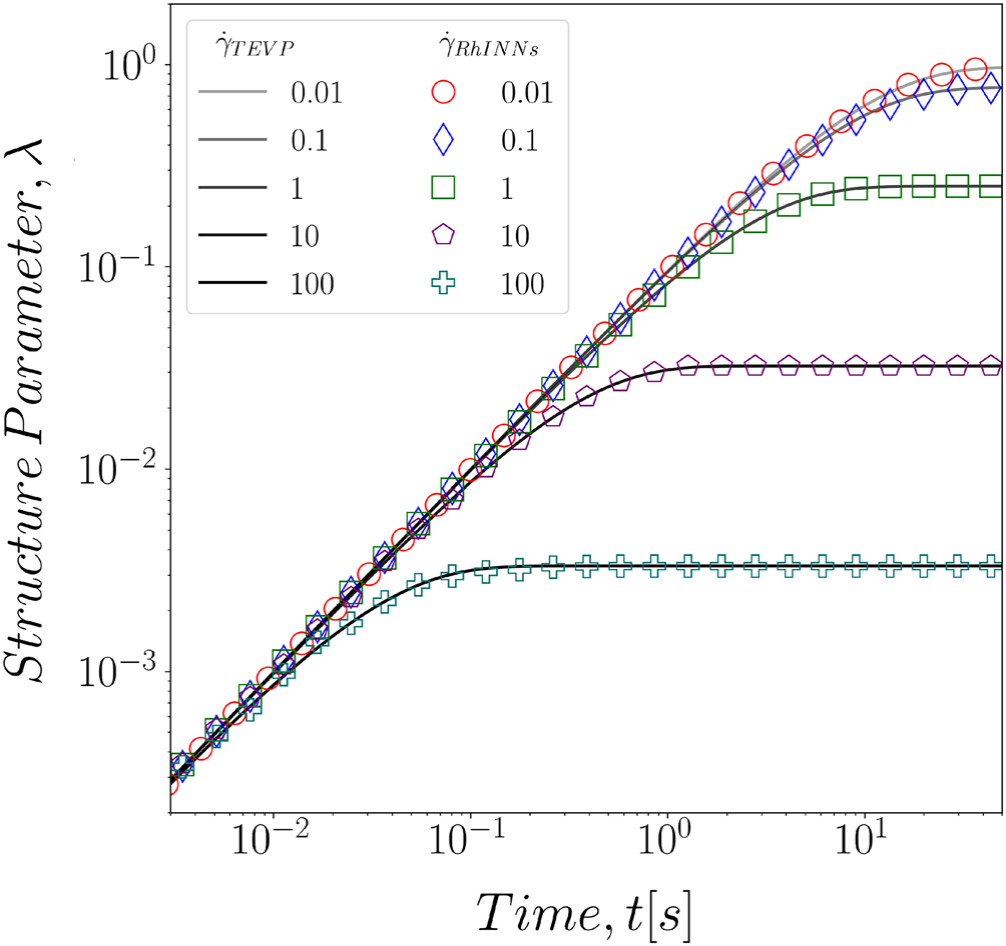
RhINNs predictions of the time evolution for structure parameter based on values of the time constants learned in one set of simple shear experiment. Here $G = 800\,[\text {Pa}]$, $\sigma _y = 20\,[\text {Pa}]$, $\eta _s = 20\,[\text {Pa s}]$, and $\eta _p = 20\,[\text {Pa s}]$ are known from the material properties.

In a similar fashion, we also investigated the impact of imposed shear rate on the performance of the RhINNs in solving the inverse problem, and finding the time constants for the structure evolution, $k_+$ and $k_-$. Table 3 shows the RhINNs predictions and the actual values for five (5) different shear rate magnitudes as studied in the direct problem. Evidently, the RhINNs-predicted time constants are closely tracking the actual values, with better efficiency in the intermediate range of applied shear rates. This could simply be explained by the fact that at the two extremeties, the low stress and high stress responses of the material become more dominant and thus slightly impact the overall predictions. Nonetheless, RhINNs predictions remain in the range of less than 5 percent error for all shear rates studied. Alternatively, Fig. 7 shows the time evolution of the structure parameter using RhINNs-predicted time constants for the flow curves previously seen in Fig. 4, compared to ground solution of the same parameter from a TEVP model. In these curves, RhINNs is solving for the time evolution of the microstructure parameter, based on a single shear stress vs. applied shear rate flow curve.

**Table 3:** RhINNs predicted values for $k_+$ and $k_-$ in different values of simple shear rate. For all cases $G = 800\,[\text {Pa}]$, $\sigma _y = 20\,[\text {Pa}]$, $\eta _s = 20\,[\text {Pa s}]$, and $\eta _p = 20\,[\text {Pa s}]$ are known from the material properties.

	Actual value	Predicted value				
		${\dot{\gamma }} = 0.01$	${\dot{\gamma }} = 0.1$	${\dot{\gamma }} = 1$	${\dot{\gamma }} = 10$	${\dot{\gamma }} = 100$
$k_-$	0.300	0.316	0.299	0.300	0.283	0.287
% Error	–	5.41	0.04	0.04	5.63	4.33
$k_+$	0.100	0.100	0.100	0.100	0.096	0.096
% Error	–	0.03	0.00	0.05	3.63	3.68

One of most important factors contributing to the performance of our proposed RhINNs is the sensitivity of the method to noisy data. Indeed most of the experimental results are naturally associated with some level of noise, due to experimental artifacts and unknown variables affecting the results. To ensure applicability of RhINNs to real-world experimental data, we investigated the effect of noisy data on parameter prediction of RhINNs in an inverse solution. We are considering one of the cases presented in Table 3 with shear rate of ${\dot{\gamma }}=0.1\,[1/\text {s}]$ in a start-up of a flow. We intentionally introduce different levels of noise based on uncorrelated Gaussian noise process to the data at hand. Table 4 represents the results of the predicted coefficients for the structure evolution, $k_+$ and $k_-$. Upon addition of 5% noise to the data, the predictions remain in very good agreement with the ground solution. This further confirms that the proposed RhINNs algorithm is not compromised by the noisy data and the predictions stay realistic and explanatory of the material under question.

**Table 4:** RhINNs predicted values for $k_+$ and $k_-$ in a start-up of a flow with shear rate of ${\dot{\gamma }}=0.1\,[1/\text {s}]$ with noisy data. For all cases $G = 800 [\text {Pa}]$, $\sigma _y = 20\,[\text {Pa}]$, $\eta _s = 20\,[\text {Pa s}]$, and $\eta _p = 20\,[\text {Pa s}]$ are known from the material properties.

	Actual value	Predicted value with noise of			
		0%	1%	2.5%	5%
$k_-$	0.300	0.299	0.295	0.289	0.284
% Error	–	0.04	1.74	3.61	4.65
$k_+$	0.100	0.100	0.097	0.094	0.092
% Error	–	0.00	1.01	5.05	7.69

We also interrogated the performance of our inverse RhINNs methodology to determine the entire list of material properties/model parameters from a limited number of experiments. To do this, we have provided the time evolution of the shear stress response of a TEVP fluid to our RhINNs platform and ask for the model to predict six (6) model parameters involved: the two time constants for the kinetics of structure formation and break-up, the elastic modulus, yield stress, and the background and plastic viscosities. Table 5 represents the actual against RhINNs-predicted values of all of these material properties provided the simple shear rate flow curves. The predictions are in excellent agreement with the actual values.

**Table 5:** RhINNs-predicted values for all coefficients based on 10 different experiments in a start-up of a flow with shear rates ranging between 0.1 and 1 [1/s].

	$k_-$	$k_+$	*G*	$\sigma _y$	$\eta _s$	$\eta _p$
Actual value	0.30	0.10	5.50	0.50	5.00	1.00
Predicted value	0.309	0.095	5.500	0.506	4.991	1.115
% Error	3.05	4.73	0.12	1.26	0.18	11.49

**Figure 8:**
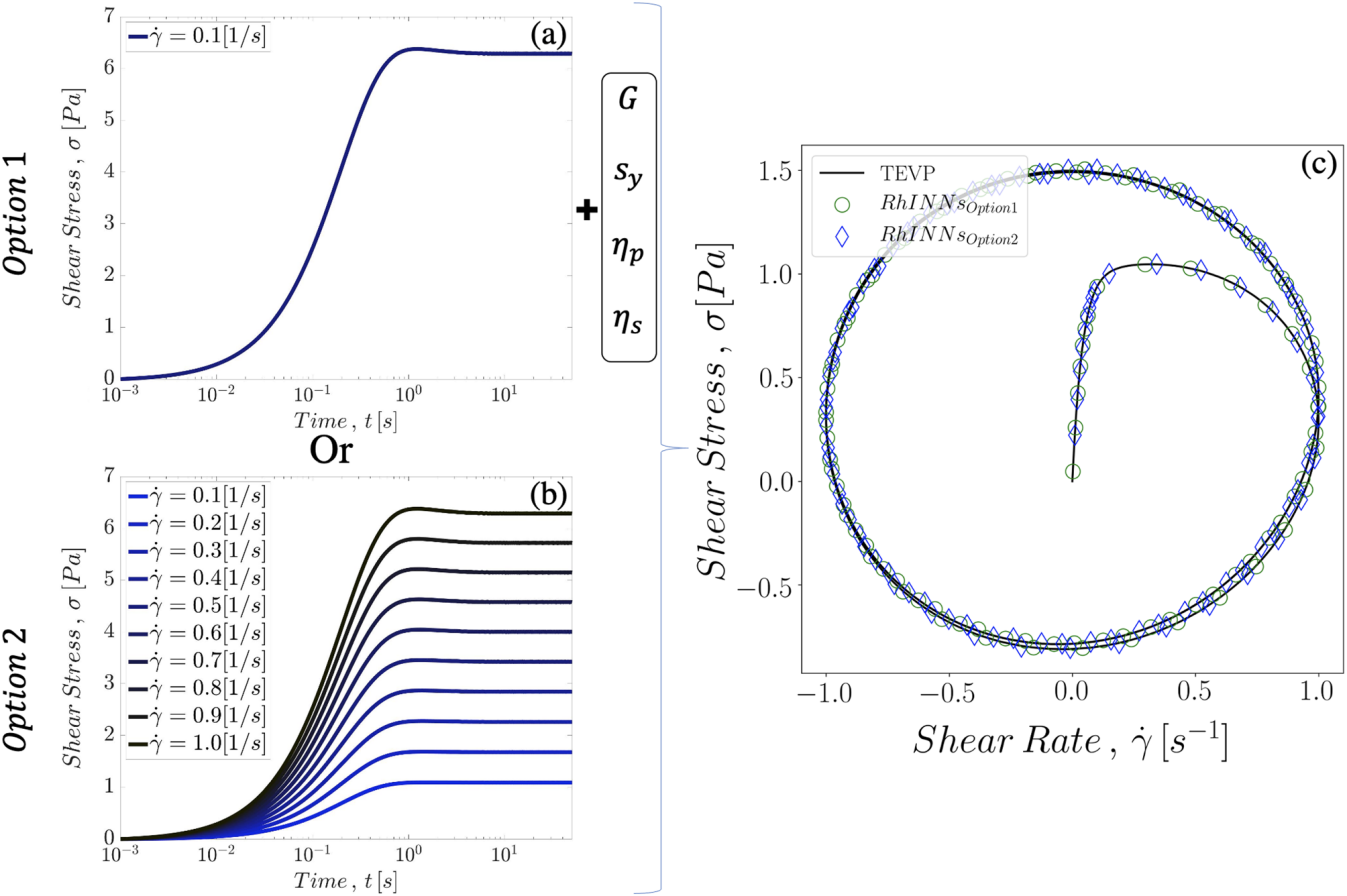
Combination of data driven discovery and data driven solution methodologies. First option provides the elastoviscopalstic properties and the results of a simple shear rate experiment, and the second option is only informed by ten different flow curves at constant shear rates without any prior knowledge of material parameters. In both options, these information are utilized to learn the hidden rheology, and subsequently predict the stress response of the TEVP fluid to a LAOS protocol.

As demonstrated in Figs. 5, 6 and Table 5, the RhINNs platform accurately predicts the time evolution of the shear stress response of a thixotropic fluid under different flow protocols having the material properties or vise versa. Hence, combining the forward and inverse solutions, i.e. data-driven solution and discovery, one can recover the material properties through a series of simple experimental protocols followed by accurate prediction of the material behavior under a different more complex flow. This is investigated here by evaluating the possibility of predicting the stress behavior of a TEVP fluid under complex shear rate protocol, given its stress response to a simple shear experiment. Figure 8 presents the RhINNs predictions following two different provided data sets for a TEVP fluid under LAOS protocol: i. elastic modulus, yield stress, background and plastic viscosities are known, as well as the time evolution of the shear stress for a single applied shear rate, and ii. no information is available for the material, but shear stress responses are available for ten (10) different applied shear rates. It should be mentioned that both of these scenarios present realistic experimental protocols. For instance, from a single flow protocol, one can measure the yield stress value, the terminal Newtonian viscosity at the highest shear rates, and the background viscosity knowing the chemical nature of the background fluid. Alternatively, one may have virtually no information about these material properties, but able to run a series of simple shear rate protocols. Regardless of the available information, the RhINNs architecture provides an excellent prediction compared to ground solution of a TEVP fluid. In both of these settings, a data driven discovery RhINNs is in serial with a data driven solution RhINNs (Fig. 8).

## Conclusion

In this work, we introduced and studied the performance of an adaptable and comprehensive data-driven algorithm for constitutive meta-modeling of complex fluids with respect to their rheological behavior. The proposed Rheology-Informed Neural Networks, RhINNs, is capable of taking advantage of NN versatility in solving constitutive equations for both direct and inverse problems. In the direct problems, the RhINNs can be used as an alternative method for solution of coupled ODEs with excellent accuracy and efficiency. This is particularly of interest with respect to complex rheological constitutive models that are commonly challenging to be implemented within CFD platforms of choice. In the inverse solution, referred to as data-driven discovery, the RhINNs accurately recovers the material properties and the model parameters having only a limited number of data sets and rheometric measurements. Due to presence of different timescales and different effects depending on the flow history, traditional approaches require several experimental protocols tested to find the best parameter fitting of a complex fluid model and to describe the system under question. Here and using RhINNs, we show only one (assuming we have partial information) or 10 (for a brand new material) simple start-up of a flow experimental data are sufficient to calculate the model parameters with a very good accuracy. This provides an extremely powerful platform for employing data-driven and machine learning algorithms in areas of research where often small sizes of data available prevents a meaningful predictive capability to be devised. To test the robustness of our proposed method, we showed one can easily determine the model parameters with a great accuracy, regardless of the type of experimental data at hand. We demonstrated that the incorporation of a physical intuition into the neural network architecture in the form of a constitutive model significantly improves the predictive ability of the algorithm. We also argue that even with a similar computational efficiency for the training of RhINNs compared to that of the traditional approaches, the main advantage of RhINNs methodology (and in general, similar science-based data-driven techniques) lies within reduction of the required data to determine model parameters and thus full characterization of a material with respect to any given rheological or thixotropic constitutive relation of interest. We need to stress on the fact that the goal of current work is not to provide a replacement for ODE solvers in either direct or inverse problem, but solely introducing a data driven method that can further be used as a powerful platform for integration of non-Newtonian constitutive laws of interest. It should also be noted that while inverse solution through common ODE backpropagator solvers will return a constant solution for characterization of a material, RhINNs (and other data-driven techniques) improve upon availability of more data and thus provide a more reliable characterization over time as well. RhINNs methodology introduced here can be directly used in order to significantly reduce the number of experiments required for probing different material properties and model parameters.

## Supplementary Information

Supplementary Information.
