# Supplementary material for: Rheology-Informed Neural Networks (RhINNs) for forward and inverse metamodelling of complex fluids
Source: Sci Rep. 2021 Jun 8;11:12015. doi: 10.1038/s41598-021-91518-3 (PMC8187644; doi:10.1038/s41598-021-91518-3)
Supplement: Supplementary file 1 — Supplementary Information. [file 41598_2021_91518_MOESM1_ESM.pdf]

Appendix

A Residual and Loss

Here, we are presenting the convergence plot and the residual losses for the RhINNs architecture. Figure A.1 presents the residual losses of the RhINNs training process. As can be seen from the figure, $\sim 15,000 - 20,000$ iterations are required to reach the assumed criteria. In this situation, the training process stops and the metamodel is deemed ready to be used for making predictions. It should be noted that the criteria used throughout this study is for the residual to be smaller than 10^{-7}

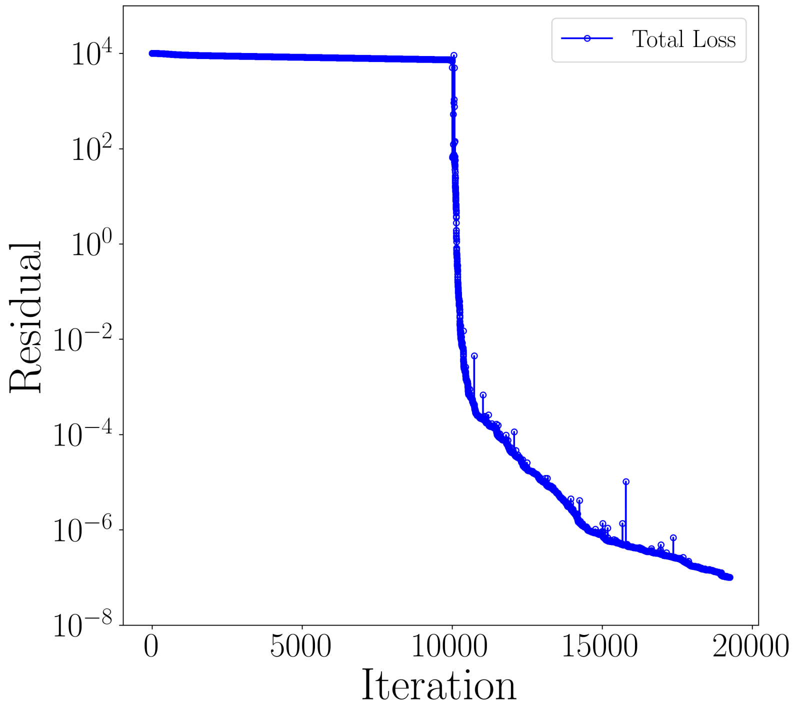

Figure A.1. *Required iterations during optimization of the loss function in the training process of RhINNs.*

B Architecture of Neural Networks

The architecture of the neural network can directly influence the performance of the framework and accuracy of the predictions made. Namely, the number of hidden layers within the NN architecture (depth), and the number of neurons per layer (width) will change the accuracy of output predictions made. Here, we use the Relative Absolute Error (RAE) as outlined in equation 1 as the measure of accuracy to compare the role of network depth and network width for our RhINNs.

$$RAE = \frac{1}{N} \sum_{n=1}^{N} \frac{|y_{actual} - y_{predicted}|}{y_{actual}} \tag{1}$$

All calculations are done based on a direct RhINNs for a start-up of a flow with shear rate of $\dot{\gamma} = 0.1[1/s]$ with TEVP model. Increasing the number of hidden layers as well as the neurons in each layer adds complexity to the NNs, however, this increase does not necessarily result in increasing the accuracy of its predictions. Adding more neurons to the NN can lead to a well-known artifact, referred to as overfitting, which in turn reduces the efficiency of the algorithm. Table 1 shows the relative error of RhINNs architectures in direct framework of a start-up of a flow with shear rate of $\dot{\gamma} = 0.1[1/s]$ for a TEVP model. In this study, widths ranging between between 5 and 20, and depths ranging between 2 and 4 are found to yield the best levels of accuracy to avoid overfitting. The architecture of the RhINNs in a direct framework is chosen to be four layers with 25 neurons in each layer. The same analysis is performed for an inverse framework of RhINNs, and in this case architecture of the RhINNs involved four layers with 50 neurons per layer.

Table 1. *The mean relative absolute error (RAE) based on different NN architectures based on their depth and width on a direct framework, TEVP model, and in a start-up of a flow.*

Width \ Depth	1	2	4	16
5	0.0203	0.00991	0.0104	0.00972
10	0.0135	0.00995	0.0101	0.00987
25	0.0108	0.0102	0.00966	0.00969
50	0.0101	0.0102	0.00988	0.00993

It should be noted that loss function is optimized using a combination of Adams optimizer and LBFG-S method together with Xavier's initialization method, while the hyperbolic tangent function is employed as the activation function throughout this work.

C Computational resources and required time

All the training and testing procedures are performed on a personal computer with 32GB RAM and i7 CPU. The highest recorded run-time for training was 12 hours with an average of two hours for each training. In other words, with a few hours of proper training, one could employ the trained RhINNs for the inverse problems to accurately recover the model parameters. In the case of noisy data, and since the procedure requires more time in handling the noise, training can be slightly longer compared to that for the clean data. It is worth mentioning that after training is performed, the predictions can be made virtually without any run time.

D Effect of initial conditions

In order to ensure a robust and rigorous training and prediction process by RhINNs, we studied six cases of different initial conditions for both shear stress and the structure parameter. We considered a direct solution in a start-up of flow with an imposed shear rate of $\dot{\gamma} = 0.1[1/s]$ using a TEVP constitutive model. The initial conditions for the shear stress are chosen to be zero as well as finite values. Since the structure parameter is inspired from the internal interaction between the microstructure of the complex fluid, we considered three different values for its initial condition: a fully structured state ($\lambda = 1$), a fully fluidized state ($\lambda = 0$), and half-structured fluid ($\lambda = 0.5$). These are experimentally relevant to different waiting times after a high-rate pre shear protocol for a gel, or any other self-assembling yield stress fluid. Figure D.1 shows the performance of RhINNs in predicting the solutions for a TEVP fluid, with respect to these studied initial conditions.

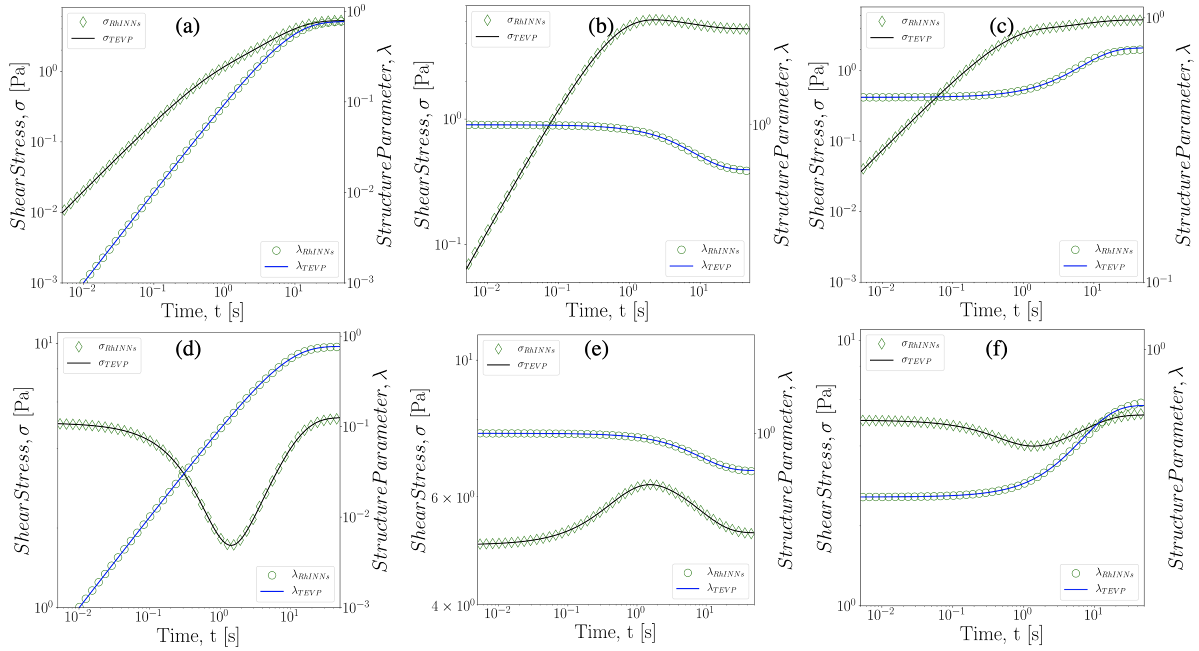

Figure D.1. *Performance of RhINNs in predicting the solution of a TEVP fluid in various initial conditions. First and second row represent the solution for a zero and non-zero initial values for the shear stress, respectively. Moreover, fully structured ($\lambda = 1$), fully fluidized ($\lambda = 0$), and half-structured fluid ($\lambda = 0.5$) states are shown in the left, middle and right columns respectively.*
